# Spontaneous splenic rupture during the recovery phase of dengue fever

**DOI:** 10.1186/s13104-015-1234-5

**Published:** 2015-07-02

**Authors:** W T T de Silva, M Gunasekera

**Affiliations:** Department of Surgery, General Hospital Kalutara, Kalutara, Sri Lanka; 23/6, 2nd Lane, Egodawatta Road, Colombo, Sri Lanka

**Keywords:** Dengue fever, Spontaneous splenic rupture

## Abstract

**Background:**

Spontaneous splenic rupture is a rare but known complication of dengue fever. Previously reported cases have occurred early during the course of the disease and most cases have led to a fatal outcome. Here we report a case of spontaneous splenic rupture in a patient with dengue fever, which occurred during the recovery phase of the illness.

**Case presentation:**

A 28-year-old Sinhalese, Sri Lankan man presented with a history of fever, myalgia and vomiting of 4 days duration. Investigations revealed a diagnosis of dengue fever with no signs of plasma leakage. He was managed in the ward as per local protocol. During the recovery phase the patient developed severe abdominal distention with circulatory failure. Radiology revealed splenic rupture with massive amounts of abdominal free fluid. The patient was resuscitated and Emergency laparotomy with splenectomy was performed. The outcome was excellent with the patient making a complete recovery.

**Conclusion:**

Although splenic rupture is a known complication of dengue fever it may be manifested late in the disease process. A high degree of suspicion should be maintained and patients must be monitored even during the recovery phase of dengue fever. Early diagnosis and intervention can prevent mortality.

**Electronic supplementary material:**

The online version of this article (doi:10.1186/s13104-015-1234-5) contains supplementary material, which is available to authorized users.

## Background

Dengue fever is the commonest arboviral illness in the world [1]. In Sri Lanka it has reached epidemic proportions with over 25,000 reported cases annually [2]. Apart from its well-known manifestation of plasma leakage leading to circulatory failure, dengue is known to lead to multiple other complications, such as myocarditis, hepatitis and neurological manifestations [3]. Spontaneous splenic rupture is a rare but known complication of dengue fever, which has been well reported in world literature [4–7]. However almost all of the reported cases have described spontaneous splenic rupture to occur early during the course of the illness. Late splenic rupture, during the recovery phase of the illness has hitherto not been reported in world literature.

## Case presentation

A 28-year-old previously healthy Sinhalese, Sri Lankan man with no known co-morbidities was admitted to Government General Hospital Kaluthara, in the Western Province of Sri Lanka with a history of fever for 4 days associated with myalgia, vomiting and anorexia. He had no haemorrhagic manifestations on admission. On examination his oral temperature was 40°C. Blood pressure was 115/86 mmHg with no significant postural hypotension. Pulse rate was 88 beats per minute. Abdomen was soft, not distended and non-tender. Lungs were clear. Investigations on admission—hemoglobin (Hb) 14.2 g/dl, packed cell volume (PCV) 41.2%, white blood cells (WBC) 1,500/mm^3^, Platelet count 68,000/mm^3^. He was managed in the medical ward as dengue fever according to Sri Lankan guidelines [2]. His platelet count continued to drop by day 5 and 6 of fever where it reached a low of 53,000/mm^3^. However he remained stable haemodynamically with no evidence of plasma leakage. His PCV remained at around 40% with adequate hydration. Dengue IgM and IgG antibodies were positive, confirming the diagnosis. By day 7, his fever settled and his platelet count started to rise and reached a level of 68,000/mm^3^ with stable PCV and haemodyanamics.

**Figure 1:**
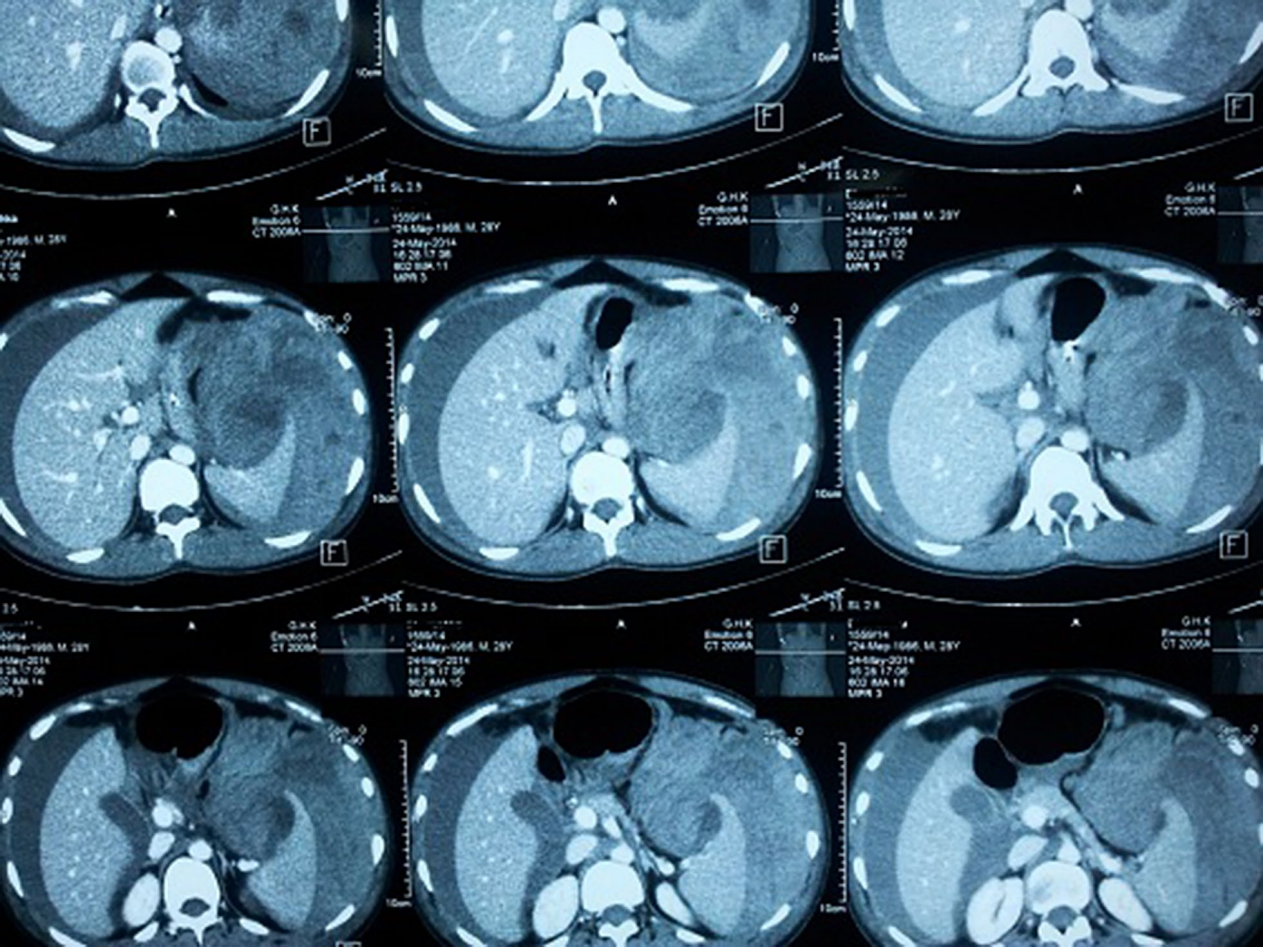
Contrast enhanced computed tomography scan of the abdomen—free fluid in peritoneal cavity with splenic laceration.

**Figure 2:**
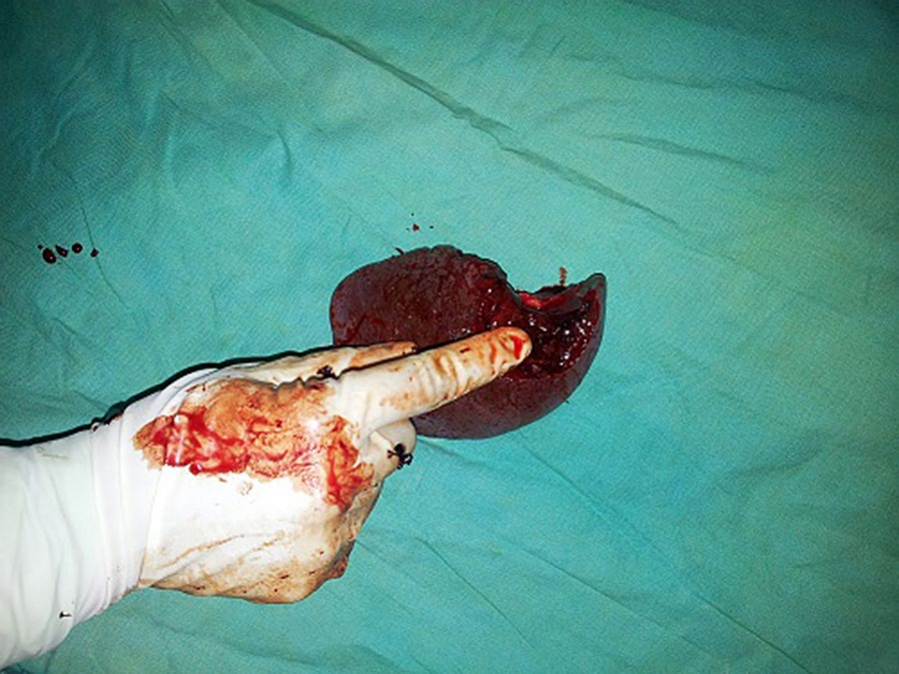
Post-splenectomy—splenic laceration close to upper pole.

On day 8, despite being afebrile, he complained of severe generalized abdominal pain. Examination revealed a distended, severely tender abdomen. He was clinically pale. His blood pressure dropped to 80/60. Pulse rate 140 bpm. Investigations—Hb 8.8 g/dl, PCV 26%, WBC 4,000/mm^3^, platelets 90,000/mm^3^. His coagulation profile was normal. A concealed bleed was suspected and the patient was transfused with two pints of packed red cells. An ultrasound scan of the abdomen at that time revealed a large amount of free fluid in the pelvis and Morrison’s pouch. A contrast enhanced computed tomography (CT) scan of the abdomen was performed (Figure 1) which revealed free fluid in the peritoneal cavity with a per-splenic haematoma. The patient was taken over to the surgical ward and an emergency laparotomy was performed. There was approximately 4 l of blood in the peritoneal cavity and a 4 cm splenic laceration close to the upper pole (Figure 2). Splenectomy was done. Four pints of packed rec cells were transfused during the surgery. Post Op day 1 the patient was haemodynamically stable. Hb 11.5 g/dl, PCV 35, WBC 14,000/mm^3^, platelets 183,000/mm^3^. The splenic histology was normal. The patient was discharged home 5 days following surgery.

## Discussion

Splenic rupture could be either post-traumatic or non-traumatic. Non-traumatic, spontaneous splenic rupture refers to one which occurs in a histologically normal spleen [8]. Many infections are known to lead to spontaneous splenic rupture, including infectious mononucleosis, malaria, typhoid, varicella, infective endocarditis, Q-fever, influenza, aspergillosis and dengue. In Sri Lanka there has been one previously published case of spontaneous splenic rupture following dengue fever [9]. When reading through all the published articles, it was evident that all previously reported cases of post-dengue spontaneous splenic rupture occurred during the acute stage of the illness, either during the viraemic stage or the critical phase (Table 1). There have been no previously documented cases of this manifestation occurring in the recovery phase of the illness. It is hypothesized that a combination of coagulation factors and severe thrombocytopenia lead to this phenomenon, however the exact mechanism of splenic rupture in dengue is not clear. The fact that this occurred in our patient during the recovery phase, where the platelet count was rising and normal coagulation profile, contradicts both theories. The splenic histology of our patient was also unremarkable rejecting any structural damage to the spleen leading to rupture. A possible mechanism which could be postulated is severe splenic congestion leading to laceration and subcapsular hematoma formation. Timely diagnosis and intervention lead to complete recovery of our patient, which is fortunate compared to most other reported cases which had a fatal outcome [11].

**Table 1:** Published case reports of spontaneous splenic rupture complicating dengue fever with timing of event

Case report	Timing of splenic rupture
Imbert et al. [4]	Day 4
Pungjitprapai and Tantawichien [5]	Day 5
Redondo et al. [6]	Day 5
Miranda et al. [10]	Day 2
Bhaskar and Moorthy [7]	Day 4

## Conclusion

Spontaneous splenic rupture although being a known complication of dengue fever, should be suspected at any phase during the disease process. Monitoring dengue patients should continue beyond the recovery phase and a high degree of suspicion should be made if signs and symptoms suggest splenic rupture. Early diagnosis and intervention will be lifesaving (Additional file 1).

## Consent

Written informed consent was obtained from the patient for publication of this Case Report and any accompanying images. A copy of the written consent is available for review by the Editor-in-Chief of this journal.

## Additional file

Additional file 1: CARE checklist.

## Abbreviations

Hb: hemoglobin
PCV: packed cell volume
HCT: haematocrit
WBC: white blood cells
IgM: immunoglobulin M
IgG: immunoglobulin G
CT: computed tomography
